# Predictive value of *in vitro* antimicrobial susceptibility testing for outcomes of *Burkholderia cepacia* complex infection: a single-center retrospective study

**DOI:** 10.3389/fmed.2026.1878930

**Published:** 2026-08-18

**Authors:** Binrong Qin, Ruirui Ma, Wenjie Cui, Rongxiang Feng, Rongqi Lu, Yun Wu, Xinyi Zhou, Ling Qin, Yali Liu

**Affiliations:** 1 Department of Laboratory Medicine, Peking Union Medical College Hospital, Chinese Academy of Medical Sciences & Peking Union Medical College, Beijing, China; 2 Graduate School, Peking Union Medical College, Chinese Academy of Medical Sciences, Beijing, China; 3 Department of Laboratory Medicine, Nanjing Drum Tower Hospital, The Affiliated Hospital of Nanjing University Medical School, Nanjing University, Nanjing, China; 4 College of Medical Laboratory, Dalian Medical University, Dalian, China; 5 Department of Infectious Diseases, Peking Union Medical College Hospital, Chinese Academy of Medical Sciences and Peking Union Medical College, Beijing, China

**Keywords:** aging, antimicrobial susceptibility testing, *Burkholderia cepacia* complex, prognosis, risk factors

## Abstract

**Introduction:**

With the Clinical and Laboratory Standards Institute (CLSI) progressively removing interpretive breakpoints for *Burkholderia cepacia* complex (Bcc), clinicians face growing uncertainty in selecting antimicrobial therapy. This study evaluated 10-year local trends in Bcc antimicrobial susceptibility and assessed whether *in vitro* antimicrobial susceptibility testing (AST) retains independent prognostic value for clinical outcomes in the absence of contemporary standardized breakpoints.

**Methods:**

This single-center retrospective study included 47 patients treated at Peking Union Medical College Hospital between 2014 and 2024, in whom Bcc was microbiologically identified and clinically confirmed as the sole causative pathogen.

**Results:**

Among the 47 patients with monomicrobial Bcc infection (27 bloodstream and 20 respiratory tract infections), the median age was 62 years and 85% had underlying comorbidities. Over the 10-year period, Bcc isolates maintained high and stable *in vitro* susceptibility to trimethoprim-sulfamethoxazole (95.6%), minocycline (95.7%), and ceftazidime (91.1%). However, *in vitro* susceptibility to these agents was not an independent predictor of clinical outcome (all *p* > 0.05). Older age was the only independent risk factor for unfavorable clinical outcomes (Firth regression: all *p* < 0.05; Bayesian: posterior mean = −0.15, 95% CI: −0.27 to −0.06; OR = 0.86, 95% CI: 0.76 to 0.94).

**Conclusion:**

Although Bcc isolates remained highly susceptible to first-line agents, *in vitro* AST results alone were insufficient to predict clinical outcomes, which were driven predominantly by host factors, particularly advanced age. In the absence of current CLSI breakpoints, AST results should be interpreted cautiously and integrated with host risk assessment, source control, and overall clinical status rather than used in isolation.

## Introduction

*Burkholderia cepacia* complex (Bcc) is an important opportunistic pathogen responsible for severe healthcare-associated infections, particularly bloodstream and respiratory tract infections in immunocompromised and critically ill patients (1–5). Owing to its intrinsically low outer membrane permeability, active efflux systems, and other complex resistance mechanisms, Bcc demonstrates intrinsic resistance to many broad-spectrum antimicrobial agents (6–8). For many years, *in vitro* antimicrobial susceptibility testing (AST) has served as an important reference for targeted treatment after failure of empirical therapy. However, the concordance between *in vitro* susceptibility phenotypes and actual clinical outcomes *in vivo* has not been adequately validated in real-world clinical cohorts.

In recent years, treatment decision-making for Bcc infection has become increasingly challenging. The Clinical and Laboratory Standards Institute (CLSI) has progressively removed AST interpretive criteria for *Burkholderia cepacia* complex (Bcc) in successive guideline updates: disk diffusion breakpoints were removed in 2024 (M100, 34th edition) (9), necessitating a switch to MIC-based methods with increased testing costs and technical demands; all clinical breakpoints were removed in 2025 (35th edition) (10), leaving only epidemiological cutoff values (ECVs) and permitting laboratories to report only MIC values without S/I/R interpretations, placing clinicians in the predicament of having MIC results without any interpretive guidance; and finally, ECVs were completely eliminated in 2026 (36th edition) (11), and the CLSI explicitly recommended against routine susceptibility testing for Bcc, forcing clinicians to rely entirely on local epidemiological data, host factors, and clinical judgment for treatment decisions. In the absence of standardized breakpoints, clinicians may still obtain minimum inhibitory concentration (MIC) results, but the clinical significance of these values is increasingly difficult to interpret. Previous studies on outcomes of Bcc infection have largely focused on patients with cystic fibrosis, and some failed to adequately exclude mixed infections and other important confounders. More importantly, in monomicrobial clinical cohorts, conventional statistical approaches are often limited by small sample size and zero-cell problems caused by highly susceptible phenotypes, hindering robust assessment of the independent prognostic value of AST.

Against this background, clarifying the local epidemiological characteristics of Bcc infection and the determinants of clinical outcome is of substantial clinical importance for optimizing management in the absence of AST breakpoints. We therefore retrospectively analyzed a rigorously selected 10-year single-center cohort of patients with monomicrobial Bcc infection and integrated local AST surveillance data across the same period. To address small-sample and zero-cell bias, we applied Firth logistic regression and complemented this with Bayesian logistic regression using weakly informative priors to ensure robust parameter estimation. This study aimed to determine: (1) Whether *in vitro* MIC-based susceptibility results retain independent prognostic value for clinical outcomes in patients with monomicrobial Bcc infection under a stable long-term local epidemiological background; and (2) whether host baseline physiological status, including age and underlying comorbidities, plays a dominant role in determining final clinical outcomes. Our goal was to provide clinically relevant local evidence to support early risk stratification and comprehensive management of Bcc infection.

## Materials and methods

### Study design and patient selection

**Figure 1:**
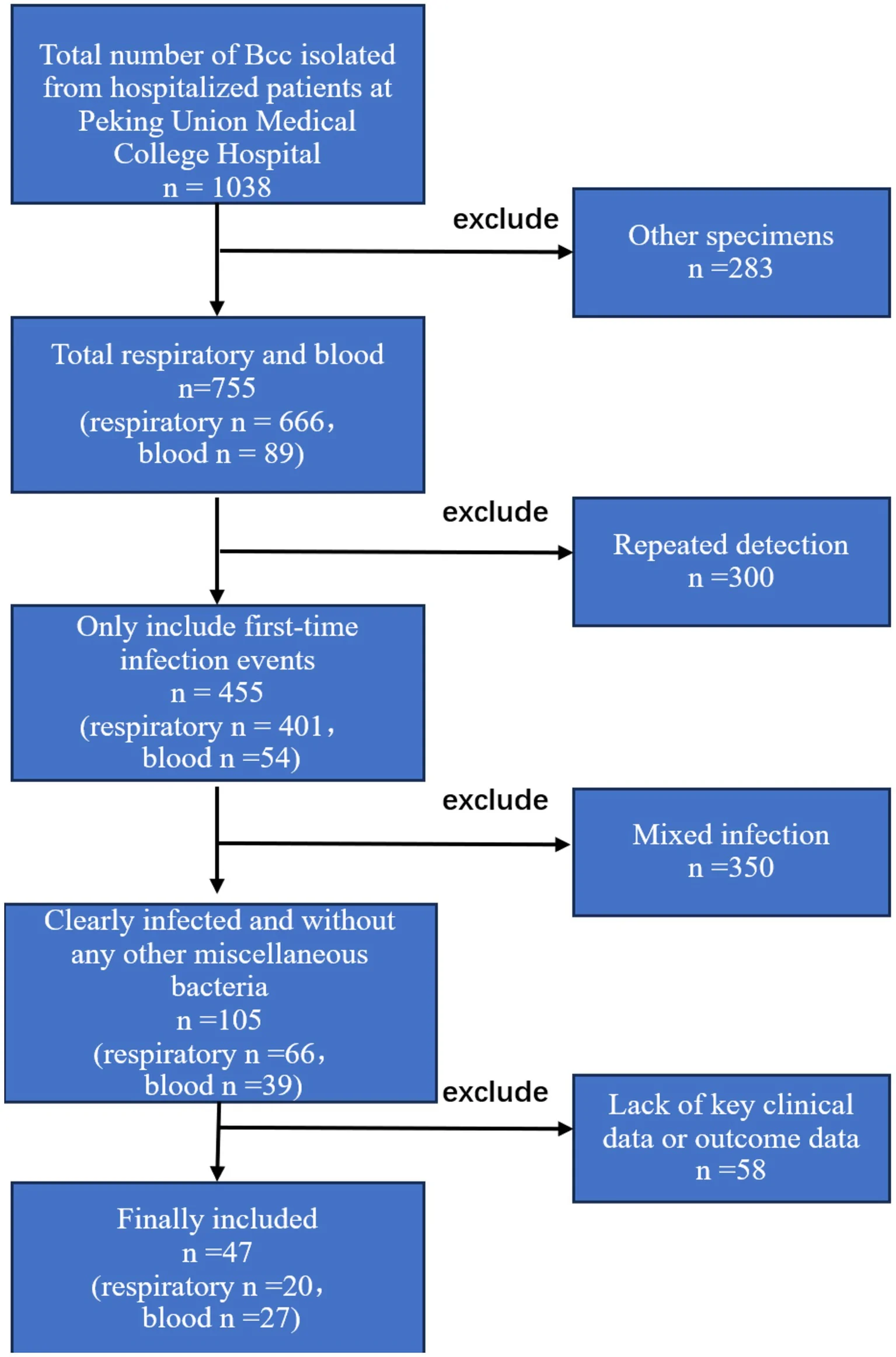
Case screening. A total of 1,038 *Burkholderia cepacia* complex (Bcc) isolates from hospitalized patients at Peking Union Medical College Hospital were initially screened. Isolates obtained from non-respiratory and non-blood specimens were excluded (*n* = 283), leaving 755 isolates from respiratory specimens (*n* = 666) and blood specimens (*n* = 89). To avoid duplicate inclusion, only first-time infection events were retained, and repeated detections were excluded (*n* = 300), resulting in 455 cases. Cases with mixed infection were further excluded (*n* = 350), leaving 105 cases with clear Bcc infection and no other miscellaneous bacteria detected. Finally, 58 cases were excluded because of missing key clinical information or outcome data. A total of 47 cases were included in the final analysis, including 20 respiratory infection cases and 27 bloodstream infection cases.

This was a single-center retrospective cohort study conducted at Peking Union Medical College Hospital. We reviewed cases from October 21, 2014 to October 17, 2024 and identified 47 patients in whom Bcc was microbiologically identified and clinically confirmed as the sole infectious pathogen and for whom complete clinical data were available (Figure 1).

The inclusion criteria were as follows:

1. Presence of signs and symptoms consistent with clinical infection;
2. Culture of Bcc from qualified clinical specimens, including blood, qualified sputum specimens, or bronchoalveolar lavage fluid, identified by the hospital clinical microbiology laboratory and considered the sole causative pathogen;
3. Availability of complete *in vitro* AST data and clinical outcome records.

For patients with multiple Bcc isolations during the study period, only the first infectious episode was included.

The exclusion criteria were:

1. Colonization or specimen contamination, defined as absence of clinical evidence of infection or explicit documentation of “colonization” without targeted anti-Bcc therapy;
2. Missing key outcome data.

### Data collection and definitions

Data were extracted using a standardized collection form, including demographic characteristics (age and sex), underlying comorbidities, invasive procedures (endotracheal intubation and central venous catheterization), infection site (bloodstream or respiratory tract), antimicrobial treatment, and clinical outcomes (12). Because of the retrospective design, detailed pharmacokinetic/pharmacodynamic parameters, antimicrobial serum concentrations, local drug exposure at infection sites, source-control timing, and standardized severity scores were not consistently available and therefore were not included in the primary analysis.

The primary endpoint was an unfavorable clinical outcome, defined as a binary variable including all-cause death after confirmed Bcc infection, or explicit documentation in the medical record of “treatment failure,” “infection worsening,” or “discharge against medical advice due to terminal condition.” Favorable clinical outcome was defined as survival with marked improvement in clinical symptoms and infection-related indicators, documented as “cured” or “improved at discharge.”

Outcome adjudication was performed independently and in a blinded manner by two investigators with backgrounds in infectious diseases and clinical microbiology. Disagreements were resolved by a third senior reviewer.

A list of all abbreviations used in this manuscript is provided in Supplementary Table S8.

### Microbiological methods

#### Species identification

All isolates were processed according to routine standard operating procedures. Initial species-level identification was performed using matrix-assisted laser desorption/ionization time-of-flight mass spectrometry (MALDI-TOF MS; Smart MS 5020, Zhuhai DL Biotech, China), and all isolates were reported as *Burkholderia cepacia* complex (Bcc).

#### AST methods and breakpoint selection

##### AST procedures

AST was routinely performed in the microbiology laboratory of Peking Union Medical College Hospital in accordance with CLSI guidance. Because this study covered isolates collected from 2014 to 2024, the AST methodology changed during the study period. Before 2021, disk diffusion (DD) was primarily used; from 2021 onward, Etest was adopted for all isolates. Both methods were performed strictly according to the manufacturers’ instructions to ensure comparability and reproducibility. Quality control was performed daily using *Escherichia coli* ATCC 25922 and *Pseudomonas aeruginosa* ATCC 27853, and all results were within the acceptable CLSI ranges.

To evaluate the impact of the methodological transition on susceptibility results, susceptibility rates obtained by DD and Etest were compared (see Results). No significant differences were observed between the two methods, supporting pooled analysis of the 10-year dataset.

##### Antimicrobial agents

The study focused on four antimicrobial agents of major clinical relevance for Bcc infection: meropenem (MEM), ceftazidime (CAZ), trimethoprim-sulfamethoxazole (TMP-SXT), and minocycline (MNO). These agents had CLSI interpretive criteria for Bcc during most of the study period and are widely used in clinical practice.

##### Breakpoint standards and data source

Because of the retrospective design, all AST results were based directly on the contemporaneous reports issued by the clinical microbiology laboratory, interpreted according to the CLSI criteria in effect at the time of testing. Across the study period (2014–2024), although the CLSI M100 document was updated annually, breakpoint values for these four agents against Bcc remained unchanged; only the disk diffusion clinical breakpoints were removed in 2024. For clarity, breakpoint values corresponding to CLSI M100, 29th edition (2019), were used as the unified reference standard in this manuscript. Laboratory AST results were categorized as susceptible (S), intermediate (I), or resistant (R) (see Supplementary Table S1). For statistical analysis, intermediate and resistant categories were combined into a single *non-susceptible* group to avoid over-stratification and loss of statistical power.

Nevertheless, because CLSI breakpoints for Bcc have subsequently been withdrawn, the susceptibility categories used in this study should be regarded as historical interpretive phenotypes rather than currently standardized clinical breakpoints. This limitation was considered when interpreting the relationship between AST results and outcomes.

### Statistical analysis

Data were analyzed using SPSS version 26.0 (IBM Corp., Armonk, NY, USA) and R version 4.4.3. Continuous variables are presented as mean ± standard deviation (SD) or median with interquartile range (IQR), depending on distribution. Categorical variables are presented as counts and percentages. In univariable analyses, categorical variables were compared using Fisher’s exact test. For analyses involving zero cells, odds ratios (ORs) and 95% credible intervals (CIs) were estimated using the Haldane-Anscombe correction. Kaplan–Meier analysis was used to estimate 30-day survival, and groups were compared with the log-rank test.

To control for potential confounders including age, sex, and comorbidities, and to address small-sample and rare-event bias, multivariable analysis was performed using Firth’s penalized logistic regression to assess the independent prognostic value of *in vitro* AST results. All tests were two-sided, and *p* < 0.05 was considered statistically significant.

To further validate the robustness of the findings given the limited number of unfavorable clinical outcomes (*n* = 11) and the small sample size, we complemented the frequentist analysis with Bayesian logistic regression using a logit link function. Regression coefficients were assigned weakly informative priors [Normal (0, 2.5)], and the intercept was assigned a Student-*t* (3, 0, 2.5) prior to stabilize parameter estimation under small-sample and sparse-event conditions. Model fitting was implemented using the R package *brms*, with 4 Hamiltonian Monte Carlo (HMC) chains, each with 4,000 iterations (2000 warm-up). Convergence was assessed using the potential scale reduction factor (Rhat) and effective sample size (ESS). Results are presented as posterior means, 95% credible intervals (CI), and odds ratios (ORs) obtained by exponentiating the log-odds coefficients.

### Ethics approval and consent to participate

This retrospective study was conducted in accordance with the ethical principles of the Declaration of Helsinki. The study protocol was reviewed and approved by the Institutional Review Board (IRB) of Peking Union Medical College Hospital (No. I-24PJ0216 and JS-2581). Due to the retrospective nature of the study and the use of de-identified clinical data, the requirement for informed consent was waived by the IRB. All patient data were anonymized prior to analysis to ensure confidentiality.

## Results

### Patient baseline characteristics

A total of 47 hospitalized patients with monomicrobial Bcc infection were included (see Supplementary Table S2). The median age was 62 years (IQR, 49–70 years). Most patients (40/47, 85%) had underlying comorbidities, of which cardiovascular disease was the most common (28/47, 60%), followed by urinary system disease (9/47, 19%), respiratory disease (8/47, 17%), autoimmune disease (4/47, 9%), and malignancy (3/47, 6%). Regarding invasive procedures, 28 patients (60%) underwent endotracheal intubation and 11 (23%) had a central venous catheter. Combination antimicrobial therapy was used in 35 patients (74%), and 22 (47%) had undergone surgery within the preceding month.

When stratified by outcome, patients with unfavorable clinical outcomes (*n* = 11) were substantially older than those with favorable clinical outcomes (*n* = 36), with a median age of 75 years (IQR, 62–83 years) versus 57 years (IQR, 37.5–68 years). All patients with unfavorable clinical outcomes had underlying comorbidities, all had cardiovascular disease, and all underwent endotracheal intubation. The proportion with central venous catheterization was also higher in the unfavorable-outcome group than in the favorable-outcome group (45% vs. 17%). These baseline findings suggested that advanced age, cardiovascular comorbidity, and invasive procedures may be associated with worse prognosis in patients with Bcc infection.

### Infection and microbiological characteristics

Among the 47 patients, 27 (57.4%) had bloodstream infection and 20 (42.6%) had respiratory tract infection.

AST results showed high overall susceptibility rates to TMP-SXT, CAZ, and MNO, at 95.6% (43/45), 91.1% (41/45), and 95.7% (44/46), respectively, whereas susceptibility to MEM was 73.9% (34/46). When the study period was divided into 2014–2019 and 2020–2024, no statistically significant changes in susceptibility rates were observed over time for any of the four agents (MEM: *p* = 1.000; TMP-SXT: *p* = 0.530; CAZ: *p* = 0.578; MNO: *p* = 1.000)(see Supplementary Table S3; Supplementary Figure S1).

Because AST methodology changed in 2021 from disk diffusion to Etest, we compared susceptibility rates between methods (see Supplementary Table S4). No statistically significant differences were found for any of the four agents (all *p* > 0.05). Specifically, MEM susceptibility was 70.6% by DD and 83.3% by Etest (*p* = 0.472); TMP-SXT susceptibility was 97.0 and 91.7% (*p* = 0.467); CAZ susceptibility was 93.9 and 83.3% (*p* = 0.286); and MNO susceptibility was 94.1 and 100.0% (*p* = 1.000). These findings support the comparability of the two AST methods and justify pooled analysis across the study period.

### Treatment and clinical outcomes

Among the 47 patients, initial empirical treatment was considered appropriate in 28 cases (59.6%), defined as coverage of at least one subsequently reported susceptible agent. All patients received antimicrobial therapy, and 35 (74.5%) were treated with combination regimens (Table 1).

**Table 1:** Clinical characteristics and outcomes of 47 infected patients.

Group	Favorable clinical outcome	Unfavorable clinical outcome	OR [95% CI]	*p*
Empirical therapy concordant with AST	21	7	0.8 [0.198–3.230]	1.000
Empirical therapy not concordant with AST	15	4		
Total	36	11		
Definitive therapy included active agent	31	9	0.726 [0.120–4.391]	0.659
Definitive therapy did not include active agent	5	2		
Total	36	11		
Underlying disease present	29	11	0.313 [0.035–2.776]	0.175
No underlying disease	7	0		
Total	36	11		

All *p*-values were calculated using two-sided Fisher’s exact test. Haldane-Anscombe correction was used to calculate OR and 95% CI.

Overall, 36 patients (76.6%) had favorable clinical outcomes and 11 (23.4%) had unfavorable clinical outcomes. Among the 28 patients whose empirical therapy was concordant with AST results, 21 (75.0%) had favorable clinical outcomes and 7 (25.0%) had unfavorable clinical outcomes. Among the 19 patients whose empirical therapy was not concordant with AST results, 15 (78.9%) had favorable clinical outcomes and 4 (21.1%) had unfavorable clinical outcomes. This difference was not statistically significant (OR, 0.8; 95% CI, 0.198–3.230; *p* = 1.000), suggesting that concordance between initial empirical treatment and AST results was not associated with clinical outcome.

Similarly, among the 40 patients whose final treatment regimen included at least one susceptible agent, 31 (77.5%) had favorable clinical outcomes and 9 (22.5%) had unfavorable clinical outcomes, whereas among the 7 patients whose final regimen did not include a susceptible agent, 5 (71.4%) had favorable clinical outcomes and 2 (28.6%) had unfavorable clinical outcomes. Again, the difference was not statistically significant (OR, 0.726; 95% CI, 0.120–4.391; *p* = 0.659).

Among the 40 patients with underlying comorbidities, the unfavorable clinical outcome rate was 27.5% (11/40), whereas no unfavorable clinical outcomes occurred among the 7 patients without documented comorbidity. Although this difference did not reach statistical significance (OR, 0.313; 95% CI, 0.035–2.776; *p* = 0.175), the trend suggests that underlying disease burden may be associated with worse prognosis; however, this should be interpreted cautiously given the small number of patients without comorbidity.

### Univariable analysis of AST results and prognosis

**Figure 2:**
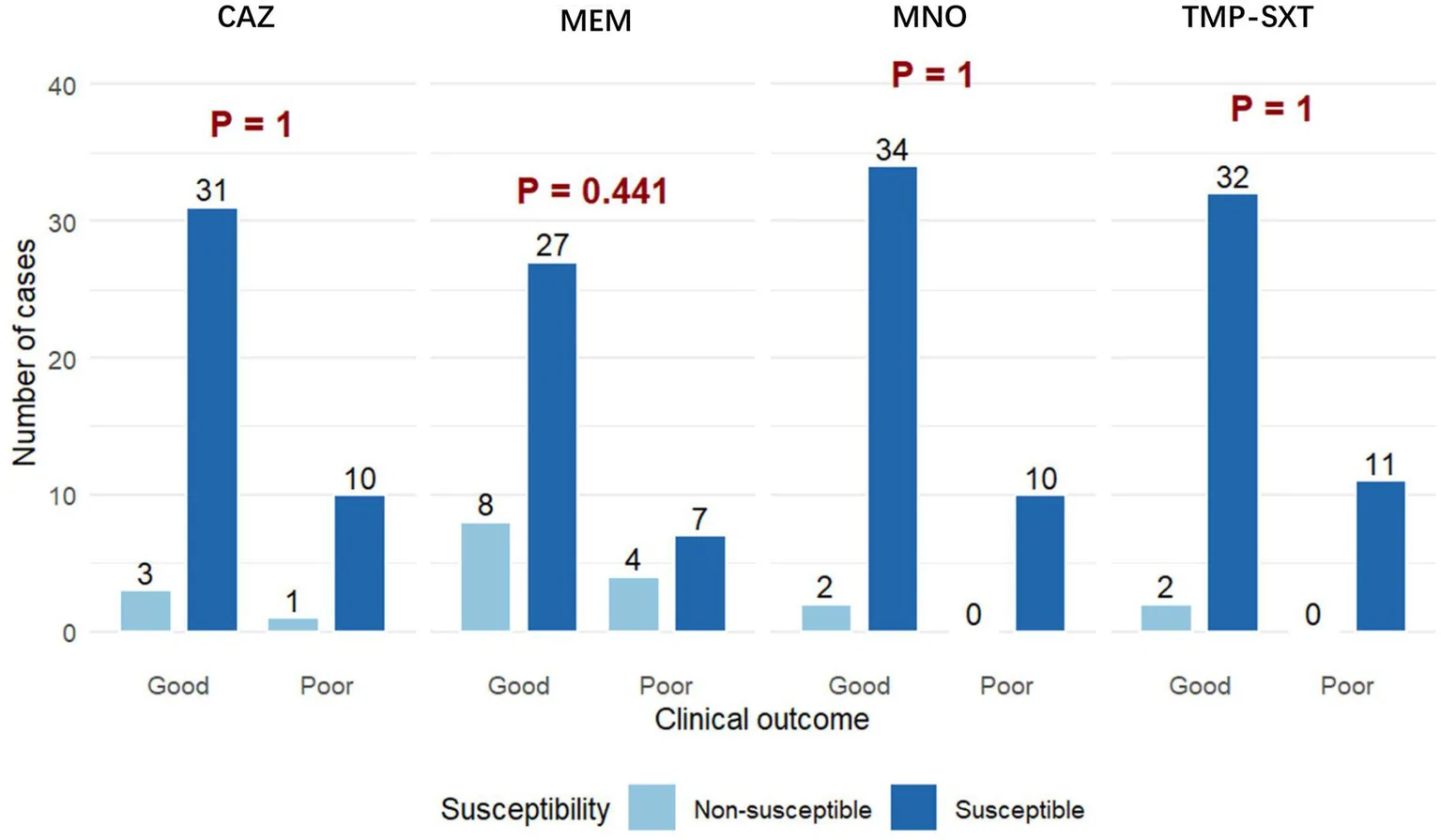
Clinical outcomes by antibiotic susceptibility. No significant association between susceptibility and prognosis was found for CAZ, MEM, MNO, or TMP-SXT (all *p* > 0.05).

**Figure 3:**
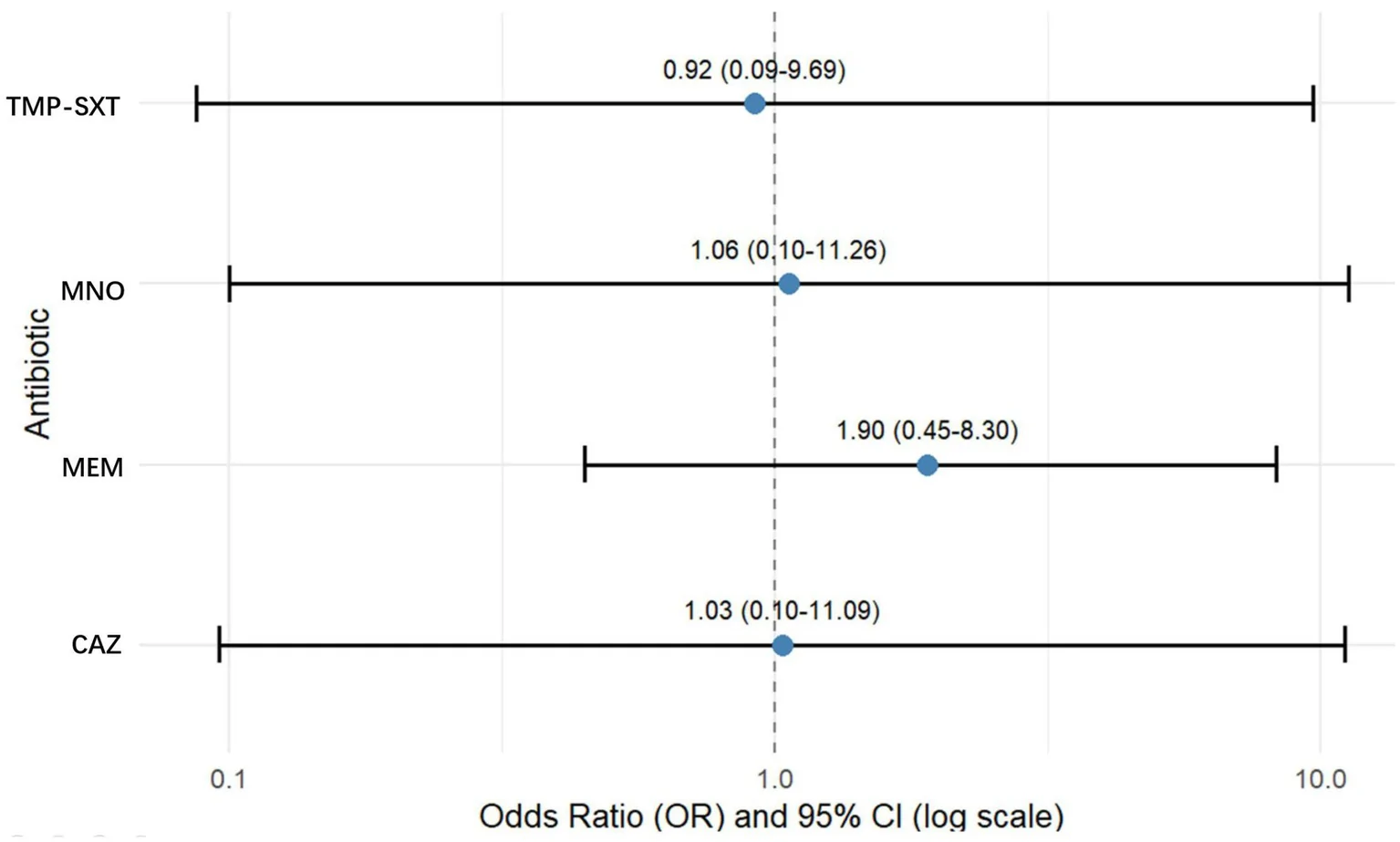
Association between antimicrobial non-susceptibility and unfavorable clinical outcomes. Odds ratios (with 95% CIs) for TMP-SXT (0.92 [0.09–9.69]), MNO (1.06 [0.10–11.26]), MEM (1.90 [0.45–8.30]), and CAZ (1.03 [0.10–11.09]) all cross 1, indicating no significant association. Haldane-Anscombe correction was applied for TMP-SXT and MNO due to zero events in non-susceptible groups.

Fisher’s exact test showed no statistically significant association between *in vitro* susceptibility and clinical outcome for any of the four antimicrobial agents studied (MEM, CAZ, TMP-SXT, and MNO; all *p* > 0.05), and all 95% CIs for ORs included 1 (Table 2; Figures 2, 3).

**Table 2:** Fisher’s Exact Test of the relationship between *in vitro* AST results and prognosis for *Burkholderia cepacia* complex.

Drug	AST result	Unfavorable clinical outcome (*n*)	Favorable clinical outcome (*n*)	Total (*n*)	OR	95% CI	*p*
MEM	Non-susceptible	4	8	12	1.899	0.448–8.304	0.441
	Susceptible	7	27	34			
CAZ	Non-susceptible	1	3	4	1.033	0.096–11.085	1.000
	Susceptible	10	31	41			
TMP-SXT	Non-susceptible	0	2	2	0.917	0.087–9.686	1.000
	Susceptible	11	32	43			
MNO	Non-susceptible	0	2	2	1.061	0.100–11.260	1.000
	Susceptible	10	34	44			

AST results were unavailable for some isolates for specific agents because of (testing panel variation/technical reasons/incomplete archived records).

All *p*-values were calculated using two-sided Fisher’s exact test. For TMP-SXT and MNO, Haldane-Anscombe correction was used to calculate OR and 95% CI due to zero events in the non-susceptible group.

For MEM, the rate of unfavorable clinical outcome was numerically higher in the *non-susceptible* group than in the susceptible group (33.3% vs. 20.6%), but this difference was not statistically significant (OR, 1.899; 95% CI, 0.448–8.304; *p* = 0.441). For CAZ, the outcome distributions were similar between susceptible and *non-susceptible* groups (OR, 1.033; 95% CI, 0.096–11.085; *p* = 1.000).

The *non-susceptible* groups for TMP-SXT and MNO were extremely small (2 cases each), with no unfavorable clinical outcomes observed. To reduce estimation bias related to zero cells, the Haldane-Anscombe correction was applied. After correction, the OR was 0.917 (95% CI, 0.087–9.686; *p* = 1.000) for TMP-SXT and 1.061 (95% CI, 0.100–11.260; *p* = 1.000) for MNO, still indicating no significant association between AST result and prognosis.

**Figure 4:**
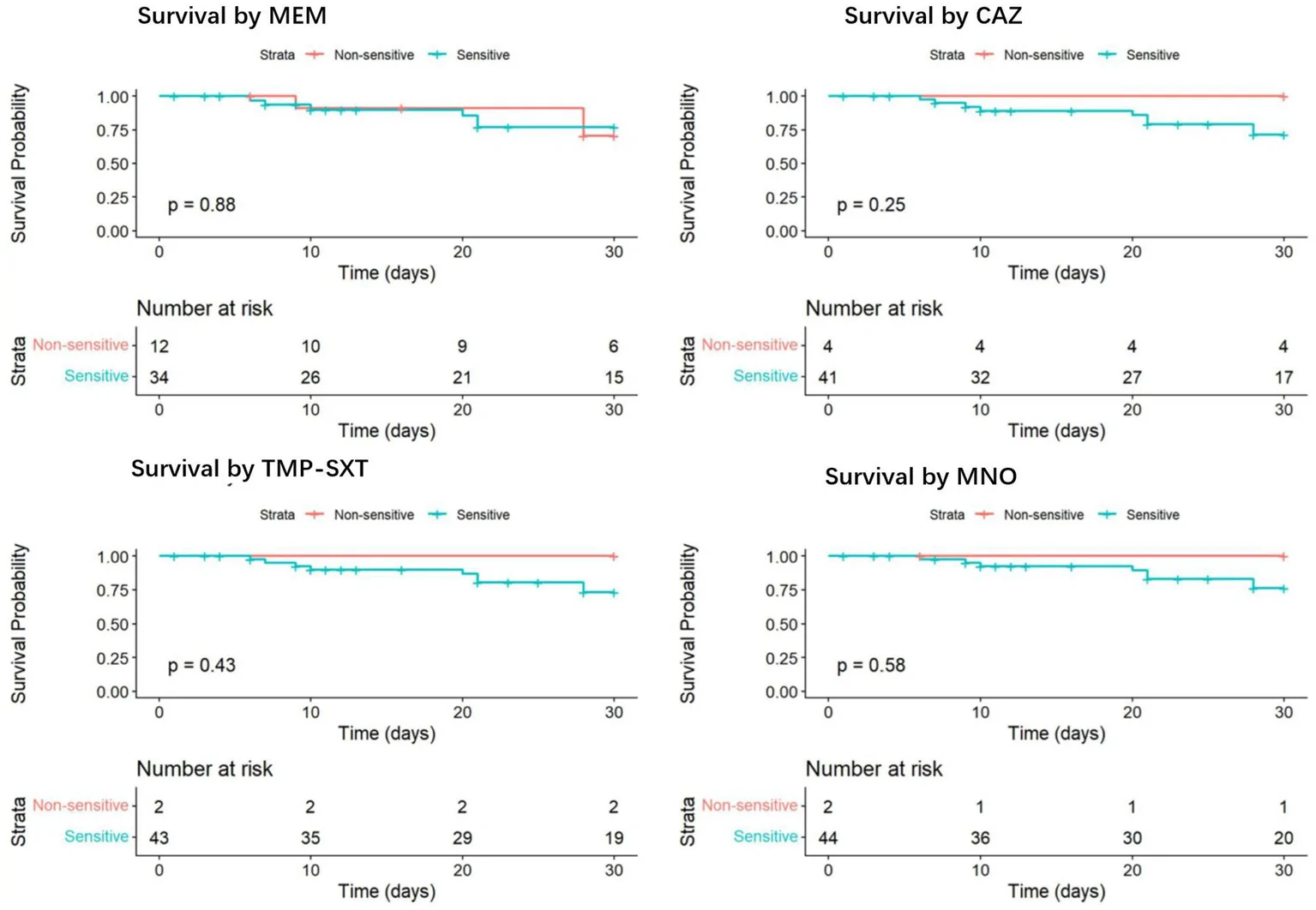
Kaplan–Meier survival analysis of 30-day outcomes by antimicrobial susceptibility in patients with monomicrobial *Burkholderia cepacia* complex infection. No significant differences were observed between susceptible and non-susceptible groups for MEM (log-rank *p* = 0.875), CAZ (*p* = 0.247), TMP-SXT (*p* = 0.431), or MNO (*p* = 0.585). Numbers at risk are shown below each panel.

To further assess the relationship between AST results and time-to-event outcomes, Kaplan–Meier analysis of 30-day survival was performed (Table 3; Figure 4). No significant differences in 30-day cumulative survival were observed between susceptible and non-susceptible groups for any antimicrobial agent (all log-rank *p* > 0.05). For MEM, the 30-day survival rate in the non-susceptible group (70.7%) was slightly lower than that in the susceptible group (77.1%), but the difference was not significant (*p* = 0.875). For CAZ, TMP-SXT, and MNO, the non-susceptible groups were very small and had no events, yielding estimated 30-day survival rates of 100%; however, there were still no statistically significant differences compared with susceptible groups.

**Table 3:** Comparison of 30-day cumulative survival rates by susceptibility to four antimicrobial agents.

Antimicrobial agent	AST group	Total cases (*n*)	Events (*n*)	30-Day cumulative survival rate	*p*
MEM	Susceptible	34	6	77.1%	0.875
	Non-susceptible	12	3	70.7%	
CAZ	Susceptible	41	9	71.3%	0.247
	Non-susceptible	4	0	100%	
TMP-SXT	Susceptible	43	9	73.1%	0.431
	Non-susceptible	2	0	100%	
MNO	Susceptible	44	8	75.9%	0.585
	Non-susceptible	2	0	100%	

*p*-value: Log-rank test (Mantel-Cox) comparing survival curves between susceptible and non-susceptible groups.

### Multivariable analysis of factors associated with prognosis

To identify independent predictors of unfavorable clinical outcome while controlling for potential confounders, we performed multivariable analysis using Firth logistic regression (Table 4; Supplementary Table S5). This approach is suitable for small-sample studies and can effectively handle complete or quasi-complete separation.

**Table 4:** Firth regression coefficients and statistics for *Burkholderia cepacia* complex.

Model	Variable	B-value	Standard error	Wald	*p*
MEM	Age	−0.081	0.035	5.378	0.020
	Sex (Female vs. Male)	−0.321	0.815	0.155	0.694
	Underlying Disease (Present vs. Absent)	−0.288	1.751	0.027	0.869
	MEM AST (Susceptible vs. Non-susceptible)	0.819	0.865	0.897	0.344
	Intercept	5.898	2.430	5.890	0.015
CAZ	Age	−0.074	0.035	4.568	0.033
	Sex (Female vs. Male)	−0.553	0.809	0.468	0.494
	Underlying Disease (Present vs. Absent)	−0.434	1.754	0.061	0.805
	CAZ AST (Susceptible vs. Non-susceptible)	0.027	1.292	0.000	0.984
	Intercept	6.247	2.666	5.491	0.019
TMP-SXT	Age	−0.084	0.035	5.622	0.018
	Sex (Female vs. Male)	−0.281	0.807	0.121	0.728
	Underlying Disease (Present vs. Absent)	−0.174	1.703	0.010	0.919
	TMP-SXT AST (Susceptible vs. Non-susceptible)	−0.393	2.125	0.034	0.853
	Intercept	6.892	3.010	5.242	0.022
MNO	Age	−0.095	0.041	5.514	0.019
	Sex (Female vs. Male)	−0.651	0.836	0.606	0.436
	Underlying Disease (Present vs. Absent)	0.063	1.832	0.001	0.973
	MNO AST (Susceptible vs. Non-susceptible)	−0.150	2.102	0.005	0.943
	Intercept	7.560	3.198	5.589	0.018

Each model included age, sex, comorbidity status, and the *in vitro* susceptibility result of one antimicrobial agent (MEM, CAZ, TMP-SXT, or MNO), with clinical outcome as the dependent variable (Favorable = 1, Unfavorable = 0). After adjustment for age, sex, and comorbidity, none of the four AST variables showed independent prognostic significance (all *p* > 0.05). The ORs for AST variables had wide credible intervals crossing 1, indicating uncertainty in both direction and magnitude of effect.

In contrast, age was a consistent independent risk factor across all four models. With each additional year of age, the odds of a favorable clinical outcome decreased by approximately 7–10% (OR range, 0.909–0.929; all *p* < 0.05). Sex and comorbidity status were not statistically significant in the multivariable models, although the direction of effect was broadly consistent across models.

Because only 11 patients experienced unfavorable clinical outcomes, these multivariable models should be interpreted as exploratory rather than definitive. The wide credible intervals, particularly for AST variables and comorbidity status, reflect limited statistical precision.

To further validate the robustness of our findings given the small sample size and sparse events, we performed Bayesian logistic regression. Model convergence was satisfactory, with all Rhat values equal to 1.00 and effective sample sizes (Bulk_ESS and Tail_ESS) exceeding 4,000 for all parameters, indicating negligible Monte Carlo error (see Supplementary Table S6).

Consistent with the Firth regression results, age was the only statistically significant predictor of favorable clinical outcome (posterior mean = −0.15, 95% CI: −0.27 to −0.06). Expressed as an odds ratio, each additional year of age was associated with an approximately 14% reduction in the odds of a favorable clinical outcome (OR = 0.86, 95% CI: 0.76 to 0.94), confirming that advanced age is an independent risk factor for unfavorable prognosis.

**Figure 5:**
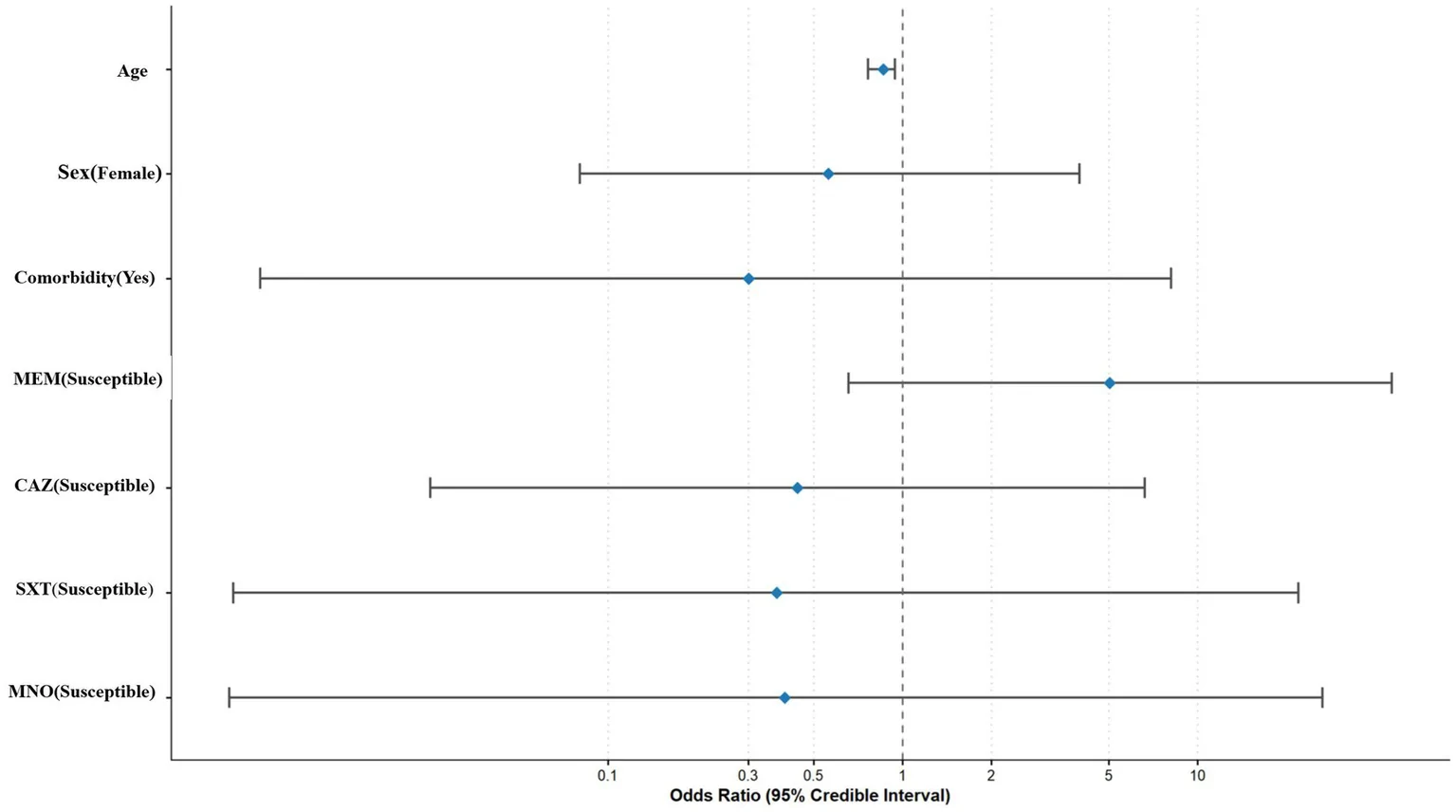
Bayesian logistic regression analysis of factors associated with clinical outcome in patients with *Burkholderia cepacia* complex infection. Forest plot showing posterior odds ratios (ORs) and 95% credible intervals derived from Bayesian logistic regression models evaluating factors associated with favorable clinical outcome. Blue diamonds indicate posterior median ORs, and horizontal lines represent 95% credible intervals. The vertical dashed line at OR = 1 indicates no association. Variables included demographic characteristics, comorbidity, and *in vitro* antimicrobial susceptibility to meropenem (MEM), ceftazidime (CAZ), trimethoprim-sulfamethoxazole (TMP-SXT), and minocycline (MNO). Credible intervals crossing 1 indicate that the association with clinical outcome was not statistically credible.

For all other covariates, the 95% credible intervals crossed the null (OR = 1), indicating no statistically significant association. The adjusted odds ratios (ORs) and 95% credible intervals (CIs) were as follows: female sex (OR = 0.56, 95% CI: 0.08–3.97), presence of underlying comorbidity (OR = 0.30, 95% CI: 0.01–8.17), MEM susceptibility (OR = 5.05, 95% CI: 0.66–45.60), CAZ susceptibility (OR = 0.44, 95% CI: 0.02–6.61), TMP-SXT susceptibility (OR = 0.38, 95% CI: 0.01–21.97), and MNO susceptibility (OR = 0.40, 95% CI: 0.01–26.57). Although some drug effect point estimates were relatively large (e.g., MEM), the extremely wide credible intervals reflect the limited sample size and underscore the uncertainty in these estimates. Overall, the *in vitro* AST results for the four agents studied did not demonstrate prognostic value independent of age (Table 5; Figure 5).

**Table 5:** Bayesian logistic regression analysis of factors associated with clinical outcome.

Variable	Estimate	Lower	Upper	OR	OR_lower	OR_upper
Age	−0.15	−0.27	−0.06	0.8607080	0.763379494	0.9417645
Sex (Female)	−0.58	−2.52	1.38	0.5598984	0.080459607	3.9749016
Comorbidity (Yes)	−1.20	−5.02	2.10	0.3011942	0.006604527	8.1661699
MEM (Susceptible)	1.62	−0.42	3.82	5.0530903	0.657046820	45.6042083
CAZ (Susceptible)	−0.82	−3.69	1.89	0.4404317	0.024972002	6.6193687
TMP-SXT (Susceptible)	−0.98	−5.23	3.09	0.3753111	0.005353525	21.9770780
MNO (Susceptible)	−0.92	−5.26	3.28	0.3985190	0.005195305	26.5757727

Estimates are posterior mean regression coefficients from the Bayesian logistic regression model and are presented on the log-odds scale. Lower and Upper indicate the lower and upper limits of the 95% credible interval for the regression coefficient, respectively. OR was calculated by exponentiating the posterior estimate, and OR_lower and OR_upper represent the corresponding 95% credible interval for the odds ratio. A positive estimate or OR > 1 indicates higher odds of a favorable clinical outcome, whereas a negative estimate or OR < 1 indicates lower odds of a favorable clinical outcome, assuming favorable clinical outcome was coded as 1. Age was modeled as a continuous variable per 1-year increase. Female sex was compared with male sex, and presence of comorbidity was compared with absence of comorbidity. Antimicrobial susceptibility variables were coded as susceptible versus non-susceptible. MEM, meropenem; CAZ, ceftazidime; TMP-SXT, trimethoprim-sulfamethoxazole; MNO, minocycline; OR, odds ratio; CI, credible interval.

## Discussion

In this rigorously selected monomicrobial cohort, we found that *in vitro* AST phenotypes did not independently predict clinical outcomes of Bcc infection in a local epidemiological setting characterized by high susceptibility to first-line antimicrobial agents. Instead, prognosis appeared to be driven predominantly by host-related factors, particularly age-related physiological decline. These findings provide clinically relevant local evidence for reassessing treatment strategies in the current era of withdrawn CLSI breakpoints for Bcc.

Our study also provides important local susceptibility data. Bcc isolates remained highly susceptible to TMP-SXT, CAZ, and MNO (95.6, 91.1, and 95.7%, respectively), whereas susceptibility to MEM was 73.9%. This profile is broadly consistent with the high TMP-SXT susceptibility reported by Lee et al. in Korea (92.8%) (13), but substantially more favorable than the low CAZ and MEM susceptibility rates reported by Özdemir et al. in Turkey (21). Chien et al. also documented substantial interspecies differences in susceptibility among Bcc isolates in Taiwan (15). These marked geographic and species-related differences underscore the importance of ongoing local surveillance when selecting empirical therapy. The progressive withdrawal of CLSI interpretive criteria for Bcc further complicates the clinical use of AST results. Although susceptibility rates in our center were stable over the 10-year period, this stability did not translate into a measurable outcome benefit. This discrepancy suggests that historical *in vitro* breakpoints may not be sufficiently linked to clinical outcomes for Bcc infection. Future breakpoint refinement, if reintroduced, should ideally incorporate outcome-linked clinical data, pharmacokinetic/pharmacodynamic exposure, infection site, and host-risk variables rather than relying solely on MIC distributions.

Beyond descriptive susceptibility patterns, the key question addressed by our study was whether AST retains independent prognostic value in Bcc infection. After correction for small-sample bias using Firth regression, we found that AST results were not independently associated with outcome, whereas age was consistently associated with worse prognosis. Bayesian sensitivity analysis produced consistent results, showing that age remained the most stable predictor, whereas AST variables had wide credible intervals and no clear independent association with outcome. This is in line with the findings of Cui et al., who reported only 40.9% concordance between *in vitro* AST and clinical outcome (16). Moore et al. further illustrated marked temporal heterogeneity in resistance phenotypes within a single patient, highlighting the difficulty of using a single AST result to predict sustained *in vivo* treatment response (17). Mechanistically, these observations support the view that static *in vitro* susceptibility results may not capture the complex, dynamic host-pathogen interactions that determine clinical outcome.

At the treatment level, we found no significant association between use of susceptible antimicrobial agents and improved outcomes. This observation is broadly consistent with prior studies (see Supplementary Table S7). Chien et al. (15) and El Chakhtoura et al. (18) similarly found that appropriate antimicrobial therapy was not independently associated with mortality reduction, whereas Lee et al. reported that early catheter removal was more strongly associated with clinical response than antimicrobial selection in catheter-related Bcc bacteremia (13). By contrast, Ku et al. (19) and Chang et al. (20) reported that inappropriate initial or definitive therapy was associated with increased mortality. These discrepancies may reflect differences in baseline patient characteristics, infection severity, source control, and definitions of treatment appropriateness.

Several biological and clinical factors may explain the lack of a direct association between AST concordance and outcome. First, host immune status may modify the effect of antimicrobial therapy, particularly in elderly or critically ill patients with reduced physiological reserve. Second, infection site may influence drug exposure, because serum-achievable concentrations do not necessarily reflect pulmonary, biofilm-associated, or device-related antimicrobial activity. Third, pharmacokinetic/pharmacodynamic variability, including tissue penetration, dosing intensity, renal function, and timing of effective therapy, may determine *in vivo* response more accurately than the categorical susceptible/non-susceptible result. Finally, source control, such as catheter removal or drainage of infected foci, may be essential for clinical improvement even when the isolate appears susceptible *in vitro*.

When AST loses its absolute interpretive value for prognosis, host factors become particularly important. In our study, older age was the only consistent independent predictor of unfavorable clinical outcome. This finding is strongly supported by larger international cohort studies, including those by El Chakhtoura et al. (*n* = 248, OR 1.06 [95% CI 1.02–1.10], *p* < 0.001, 18), Righi et al. (*n* = 46, OR 1.07 [95% CI 1.01–1.15], *p* = 0.019, 26), and Alwazzeh et al. (*n* = 151, OR 1.03 [95% CI 1.01–1.06], *p* = 0.039, 14). Özdemir et al. further showed that markers reflecting host inflammatory burden and organ dysfunction, such as neutrophil-to-lymphocyte ratio and creatinine, independently predicted short-term mortality (21). From a pathophysiological perspective, advanced age is commonly associated with immunosenescence and reduced multiorgan physiological reserve. In infections caused by opportunistic pathogens such as Bcc, the host’s immune defense and compensatory capacity may therefore be more important than the *in vitro* susceptibility phenotype itself.

Comorbidities and invasive procedures may further interact with age to influence prognosis. In our cohort, all patients with unfavorable clinical outcomes had underlying comorbidities and underwent endotracheal intubation, and all had cardiovascular disease. Although these variables were not independently significant in multivariable models, likely because of limited sample size and collinearity with age and disease severity, they may still reflect frailty, impaired host defense, and greater exposure to device-associated infection risks. Therefore, the effect of pathogen susceptibility should not be interpreted separately from host vulnerability, infection severity, and the feasibility of source control.

Based on the above findings and the latest evidence-based medicine, clinical management of Bcc infection requires a transition from MIC-centered decision-making toward comprehensive risk-based treatment. During the empirical treatment phase, based on our center’s stable decade-long antimicrobial susceptibility surveillance, trimethoprim-sulfamethoxazole combined with minocycline may be considered as a preferred local empirical option covering the vast majority of local strains in monomicrobial infections caused by Bcc. This recommendation should not be generalized to polymicrobial infections, colonization, or settings with different local susceptibility profiles. In the targeted intervention and eradication phase, although novel agents (e.g., cefiderocol) have been explored in the PERSEUS study (22), and inhaled therapy for CF patients (23) as well as the meropenem-containing eradication regimen recommended by the UK NHS (24) have shown certain clinical potential, the Cochrane systematic review (25) clearly states that there is currently insufficient high-quality randomized controlled trial (RCT) evidence to support any specific long-term antimicrobial therapy. Therefore, clinical practice should move beyond the limitation of simply focusing on MIC values and shift toward a closed-loop treatment encompassing source control, host assessment, empirical therapy, antimicrobial adjustment, and comprehensive support, so as to maximize survival outcomes in high-risk populations such as the elderly.

This study has several limitations. First, the sample size was small (*n* = 47), and only 11 patients had unfavorable clinical outcomes. This limited statistical power, produced wide confidence or credible intervals, and reduced the robustness and generalizability of the multivariable models, even though Firth regression and Bayesian analysis were used to mitigate small-sample and sparse-event bias. Second, the retrospective single-center design may have introduced selection bias and information bias. Although predefined inclusion and exclusion criteria were used to identify true monomicrobial Bcc infection and exclude colonization, contamination, and polymicrobial infection, residual bias could not be completely avoided. Third, species-level identification within the Bcc complex was not available because of routine laboratory workflow limitations, and species-specific differences in virulence and susceptibility could not be assessed. Fourth, part of the study period preceded the complete withdrawal of CLSI breakpoints for Bcc, and the AST categories used in this study therefore represent historical interpretive phenotypes rather than currently standardized clinical categories. Fifth, pharmacokinetic/pharmacodynamic parameters, antimicrobial drug exposure, tissue penetration, infection-site concentrations, detailed source-control timing, and standardized severity scores were not systematically available. These unmeasured factors may partly explain the mismatch between *in vitro* AST results and clinical outcomes. Future prospective multicenter studies with larger sample sizes, species-level identification, standardized severity assessment, PK/PD evaluation, and outcome-linked AST interpretation are needed to validate these findings.

## Data Availability

The datasets generated and/or analyzed during the current study are not publicly available due to institutional data protection policies but are available from the corresponding author on reasonable request. Requests to access these datasets should be directed to YL, liuyluijk@163.com, liuyali@pumch.cn.
