# Supplementary material for: Predictive value of in vitro antimicrobial susceptibility testing for outcomes of Burkholderia cepacia complex infection: a single-center retrospective study
Source: Front Med (Lausanne). 2026 Aug 18;13:1878930. doi: 10.3389/fmed.2026.1878930 (PMC13529570; doi:10.3389/fmed.2026.1878930)
Supplement: Supplementary file 1 [file Supplementary_file_1.docx]

**SUPPORTING INFORMATION**

**Predictive value of in vitro antimicrobial susceptibility testing for outcomes of *Burkholderia cepacia* complex infection: a single-center retrospective study**

Binrong Qin^a,b†^, Ruirui Ma^a,b†^, Wenjie Cui^a,c†^, Rongxiang Feng^a,d^, Rongqi Lu^a,b^, Yun Wu^a,b^, Xinyi Zhou^a,b^, Ling Qin^e*^, Yali Liu^a*^

^a^ Department of laboratory medicine, Peking Union Medical College Hospital, Chinese Academy of Medical Sciences & Peking Union Medical College, Beijing, China

^b^ Graduate School, Peking Union Medical College, Chinese Academy of Medical Sciences, Beijing, People’s Republic of China

^c^ Department of Laboratory Medicine, Nanjing Drum Tower Hospital, The Affiliated Hospital of Nanjing University Medical School, Nanjing University, Nanjing, China

^d^ College of Medical Laboratory, Dalian Medical University, No. 9 West Section Lvshun South Road, Dalian, China

^e^ Department of Infectious Diseases, Peking Union Medical College Hospital, Chinese Academy of Medical Sciences and Peking Union Medical College, Beijing, ^a^00730, China

^†^Binrong Qin, Ruirui Ma, Wenjie Cui contributed equally and are co-first authors.

^*^Corresponding : [qinlingpumch@126.com](mailto:qinlingpumch@126.com), liuyluijk@163.com, [liuyali@pumch.cn](mailto:liuyali@pumch.cn)

**Figure 1 Antimicrobial susceptibility rates of Bcc isolates (2014–2024).**

No significant changes were observed over time for meropenem (75.0% vs 71.4%), TMP-SXT (96.8% vs 92.9%), ceftazidime (93.5% vs 85.7%), or minocycline (93.8% vs 100.0%) (all P > 0.05).

**Table S1 CLSI Breakpoint Standards for Antimicrobial Agents Used in This Study (2014–2024)**

| Antimicrobial Agent | Disk Content | Disk Diffusion Diameter Interpretive Criteria (mm) | | | MIC Interpretive Criteria (µg/mL) | | |
| --- | --- | --- | --- | --- | --- | --- | --- |
|  |  | S | I | R | S | I | R |
| Ceftazidime | 30 µg | ≥21 | 18–20 | ≤17 | ≤8 | 16 | ≥32 |
| Meropenem | 10 µg | ≥20 | 16–19 | ≤15 | ≤4 | 8 | ≥16 |
| Minocycline | 30 µg | ≥19 | 15–18 | ≤14 | ≤4 | 8 | ≥16 |
| Trimethoprim-Sulfamethoxazole | 1.25/23.75 µg | ≥16 | 11–15 | ≤10 | ≤2/38 | – | ≥4/76 |

The breakpoint values listed above remained consistent for *Burkholderia cepacia* complex throughout the study period (2014–2024) in the relevant CLSI M100 editions, except for the removal of Disk Diffusion clinical breakpoints in 2024. For clarity and consistency in reporting, this study uniformly references the breakpoint criteria from CLSI M100 (29th edition, 2019). In the statistical analysis, the “Intermediate” (I) and “Resistant” (R) categories were combined into a “Non-susceptible” group.

**Table S2 Baseline Characteristics of Patients**

| Characteristic | favorable clinical outcome (n=36) | Unfavorable clinical outcome (n=11) | Total (n=47) |
| --- | --- | --- | --- |
| Age, median (IQR) | 57 (37.5, 68) | 75 (62, 83) | 62 (49, 70) |
| Female, n (%) | 13 (36%) | 4 (36%) | 17 (36%) |
| Male, n (%) | 23 (64%) | 7 (64%) | 30 (64%) |
| Underlying disease, n (%) | 29 (81%) | 11 (100%) | 40 (85%) |
| Cardiovascular disease | 17 (47%) | 11 (100%) | 28 (60%) |
| Respiratory disease | 5 (14%) | 3 (27%) | 8 (17%) |
| Urinary system disease | 7 (19%) | 2 (18%) | 9 (19%) |
| Oncologic disease | 2 (6%) | 1 (9%) | 3 (6%) |
| Autoimmune disease | 4 (11%) | 0 (0%) | 4 (9%) |
| Endotracheal intubation | 17 (47%) | 11 (100%) | 28 (60%) |
| Central venous catheter | 6 (17%) | 5 (45%) | 11 (23%) |
| Combination antimicrobial therapy | 26 (72%) | 9 (82%) | 35 (74%) |
| History of surgery within one month | 19 (53%) | 3 (27%) | 22 (47%) |

**Table S3 Antimicrobial Susceptibility Profile of *Burkholderia cepacia* Complex Isolates**

| Antimicrobial Susceptibility | 2014–2019  n (%) | 2020–2024  n (%) | *p* | OR [95% CI] |
| --- | --- | --- | --- | --- |
| Meropenem (n=46) |  |  | 1.00 | 1.20 [0.293–4.909] |
| Susceptible | 24 (75.0%) | 10 (71.4%) |  |  |
| Intermediate/Resistant | 8 (25.0%) | 4 (28.6%) |  |  |
| TMP-SXT (n=45) |  |  | 0.53 | 2.308 [0.134–39.783] |
| Susceptible | 30 (96.8%) | 13 (92.9%) |  |  |
| Intermediate/Resistant | 1 (3.2%) | 1 (7.1%) |  |  |
| Ceftazidime (n=45) |  |  | 0.578 | 2.417 [0.304–19.194] |
| Susceptible | 29 (93.5%) | 12 (85.7%) |  |  |
| Intermediate/Resistant | 2 (6.5%) | 2 (14.3%) |  |  |
| Minocycline (n=46) |  |  | 1.00 | – |
| Susceptible | 30 (93.8%) | 14 (100.0%) |  |  |
| Intermediate/Resistant | 2 (6.2%) | 0 (0.0%) |  |  |

All *p*-values were calculated using two-sided Fisher's exact test.

**Table S4 Antimicrobial Susceptibility Profile of *Burkholderia cepacia* Complex Isolates by Testing Method**

| Antimicrobial Susceptibility | Disk Diffusion  n (%) | Etest  n (%) | *p* | OR [95% CI] |
| --- | --- | --- | --- | --- |
| Meropenem |  |  | 0.472 | 0.48 [0.089–2.596] |
| Susceptible | 24 (70.6%) | 10 (83.3%) |  |  |
| Intermediate/Resistant | 10 (29.4%) | 2 (16.7%) |  |  |
| TMP-SXT |  |  | 0.467 | 2.909 [0.167–50.554] |
| Susceptible | 32 (97.0%) | 11 (91.7%) |  |  |
| Intermediate/Resistant | 1 (3.0%) | 1 (8.3%) |  |  |
| Ceftazidime |  |  | 0.286 | 3.1 [0.385–24.952] |
| Susceptible | 31 (93.9%) | 10 (83.3%) |  |  |
| Intermediate/Resistant | 2 (6.1%) | 2 (16.7%) |  |  |
| Minocycline |  |  | 1.00 | – |
| Susceptible | 32 (94.1%) | 12 (100.0%) |  |  |
| Intermediate/Resistant | 2 (5.9%) | 0 (0.0%) |  |  |

All *p*-values were calculated using two-sided Fisher's exact test.

**Table S5 Firth Regression Analysis of Factors Influencing Prognosis in Burkholderia cepacia Complex Infections**

| Variable | Category | MEM Model | CAZ Model | TMP-SXT Model | MNO Model |
| --- | --- | --- | --- | --- | --- |
| Age | Continuous | 0.923 (0.862–0.988)* | 0.929 (0.867–0.994)* | 0.920 (0.858–0.986)* | 0.909 (0.839–0.984)* |
| Sex | Female vs. Male | 0.725 (0.147–3.583) | 0.575 (0.118–2.807) | 0.755 (0.155–3.674) | 0.522 (0.101–2.685) |
| Underlying Disease | Present vs. Absent | 0.750 (0.024–23.185) | 0.648 (0.021–20.151) | 0.841 (0.030–23.656) | 1.065 (0.029–38.641) |
| In Vitro AST Result | Susceptible vs. Non-susceptible | 2.268 (0.416–12.355) | 1.027 (0.082–12.924) | 0.675 (0.010–43.450) | 0.861 (0.014–52.955) |

Data are presented as OR (95% CI); * indicates *P* < 0.05. Models were adjusted using Firth regression to correct for small sample bias and separation issues.

**Table S6 Bayesian logistic regression analysis of factors associated with clinical outcome in patients with Burkholderia cepacia complex infection**

|  | Estimate | Est.Error | l-95%CI | u-95%CI | Rhat | Bulk_ESS | Tail_ESS |
| --- | --- | --- | --- | --- | --- | --- | --- |
| Intercept | 13.75 | 4.70 | 5.29 | 23.71 | 1.00 | 6130 | 5595 |
| age | -0.15 | 0.05 | -0.27 | -0.06 | 1.00 | 5284 | 4271 |
| Sex | -0.58 | 0.99 | -2.52 | 1.38 | 1.00 | 8953 | 5917 |
| Comorbidity | -1.20 | 1.82 | -5.02 | 2.10 | 1.00 | 8388 | 5427 |
| MEM | 1.62 | 1.09 | -0.42 | 3.82 | 1.00 | 8660 | 5801 |
| CAZ | -0.82 | 1.42 | -3.69 | 1.89 | 1.00 | 8861 | 5936 |
| TMP-SXT | -0.98 | 2.14 | -5.23 | 3.09 | 1.00 | 7820 | 5816 |
| MNO | -0.92 | 2.16 | -5.26 | 3.28 | 1.00 | 8844 | 6061 |

Estimates are posterior mean regression coefficients from the Bayesian logistic regression model and are presented on the log-odds scale. A positive estimate indicates higher odds of a favorable clinical outcome, whereas a negative estimate indicates lower odds, assuming favorable outcome was coded as 1. Est.Error represents the posterior standard deviation. l-95%CI and u-95%CI indicate the lower and upper limits of the 95% credible interval, respectively. Rhat values close to 1.00 and adequate Bulk_ESS and Tail_ESS values indicate acceptable Markov chain Monte Carlo convergence and sampling efficiency. Age was modeled as a continuous variable per 1-year increase. Female sex was compared with male sex, and presence of comorbidity was compared with absence of comorbidity. Antibiotic susceptibility variables were coded as sensitive versus non-sensitive. MEM, meropenem; CAZ, ceftazidime; TMP-SXT, trimethoprim-sulfamethoxazole; MNO, minocycline; ESS, effective sample size.

**Table S7 Summary of Current Literature on This Topic**

| Author (Year), Country | Study Design | Sample Size | Key Findings |
| --- | --- | --- | --- |
| Mondal et al. (2025), India (1) | Retrospective cohort study + systematic review | 124 Bcc bloodstream infections | Multidrug-resistant (MDR) phenotype not significantly associated with mortality (*P* = 0.18). Multivariate analysis identified qSOFA score, ICU admission, and MODS as independent predictors of mortality. |
| Cui et al. (2025), China (2) | Methodological comparative study | 44 Bcc infections | Concordance between in vitro AST and clinical outcomes was only 40.9%, highlighting the limited value of AST in guiding treatment decisions. |
| Özdemir et al. (2023), Turkey (3) | Retrospective single-center study | 87 Bcc infections | In vitro AST results not significantly correlated with 14-day and 28-day mortality (*P* > 0.05). Host inflammatory/organ function markers (NLR, creatinine, PCT) were independent predictors of 14-day and 28-day mortality. |
| Hedvat et al. (2020), USA (4) | Retrospective study (conference abstract) | 68 Bcc infections | No difference in treatment failure between monotherapy and combination therapy (*P* = 0.947); multivariate analysis showed only SOFA score associated with treatment failure (OR 1.43). |
| Lee et al. (2020), South Korea (5) | Multicenter retrospective study | 216 Bcc bacteremia (non-CF) | Appropriate antimicrobial therapy not associated with mortality (*P* = 0.55); independent risk factors for 30-day mortality: female sex, liver cirrhosis, septic shock, and catheter-related infection; early catheter removal critical. |
| El Chakhtoura et al. (2017), USA (6) | Nationwide retrospective cohort study | 248 Bcc bloodstream infections | Appropriate antimicrobial therapy not associated with mortality (*P* = 0.071). Age and Pitt bacteremia score independent predictors of 30-day mortality. |
| Chang et al. (2022), China (7) | Retrospective single-center study | 195 Bcc bacteremia (non-CF) | Although inappropriate definitive therapy associated with mortality, no single treatment regimen associated with improved mortality. Multivariate analysis: Pitt bacteremia score, metastatic cancer associated with 14-day mortality. |
| Moore et al. (2024), UK (8) | Case report (longitudinal analysis) | 1 CF patient, 11 isolates over 63 months | 11 isolates of *B. cenocepacia* from same patient over 63 months showed 11 distinct resistotypes; susceptibility results not correlated with time (r² = 0.0583); >40 colonies needed to capture all resistotypes. |
| Shukla et al. (2024), UK (9) | Laboratory study | – | Antibiotics susceptible in standard AST showed significantly reduced activity in artificial sputum medium mimicking CF lung environment. |
| Gutiérrez Santana & Coria Jiménez (2024), Mexico (10) | Review (cystic fibrosis) | – | Explicitly states "lack of correlation between antimicrobial susceptibility patterns and clinical outcomes" and emphasizes need for clinical effectiveness studies to establish standardized treatment regimens. |

**Table S8 List of abbreviations**

| abbreviation | full name |
| --- | --- |
| AST | Antimicrobial Susceptibility Testing |
| ATCC | American Type Culture Collection |
| BAL | Bronchoalveolar Lavage |
| Bcc | *Burkholderia cepacia* complex |
| BMD | Broth Microdilution |
| CAZ | Ceftazidime |
| CF | Cystic Fibrosis |
| CI | Confidence Interval |
| CLSI | Clinical and Laboratory Standards Institute |
| COPD | Chronic Obstructive Pulmonary Disease |
| DD | Disk Diffusion |
| ECV | Epidemiological Cutoff Value |
| Etest | Etest (gradient diffusion method) |
| I | Intermediate |
| ICU | Intensive Care Unit |
| IQR | Interquartile Range |
| MALDI-TOF MS | Matrix-Assisted Laser Desorption/Ionization Time-of-Flight Mass Spectrometry |
| MEM | Meropenem |
| MNO | Minocycline |
| MODS | Multiple Organ Dysfunction Syndrome |
| NLR | Neutrophil-to-Lymphocyte Ratio |
| OR | Odds Ratio |
| PCT | Procalcitonin |
| QC | Quality Control |
| R | Resistant |
| S | Susceptible |
| SD | Standard Deviation |
| TMP-SXT | Trimethoprim-Sulfamethoxazole |

**Reference:**

1.	Mondal S, Mohanty NR, Pattnaik SS, Choudhary A, Aggarwal L, Patro S, Mondal S, Mohanty NR, Pattnaik SS, Choudhary A, Aggarwal L, Patro S. 2025. Multidrug resistance and clinical predictors of mortality in burkholderia cepacia complex bacteremia: Local evidence and global perspectives with a review of the literature. Cureus 17.

2.	Cui W, Lu R, Huang S, Ma R, Wang Q, Liu Y. 2025. Comparative evaluation of broth microdilution and gradient diffusion methods in burkholderia cepacia complex susceptibility testing: Implications of transitioning to ECV-based interpretation. J Antimicrob Chemother 80:2530–2540.

3.	Özdemir YE, Kaplan-Yapar B, Borcak D, Canbolat-Ünlü E, Bayramlar OF, Çizmeci Z, Kart-Yaşar K. 2023. Antimicrobial Susceptibility Profiles and Key Determinants for Mortality in Burkholderia cepacia Complex Infections. Infect Dis Clin Microbiol 5:239–250.

4.	Hedvat J, Kubin CJ, Mehta M. 2020. 1579. Burkholderia returns: Are two drugs better or back to bactrim? Open Forum Infect Dis 7:S787–S788.

5.	Lee Y-M, Park K-H, Moon C, Kim DY, Lee MS, Kim T, Choo EJ, Chong YP, Kim S-H, Kim YS, Woo JH, Chang MS. 2020. Management and outcomes of burkholderia cepacia complex bacteremia in patients without cystic fibrosis: A retrospective observational study. European Journal of Clinical Microbiology & Infectious Diseases 39:2057–2064.

6.	El Chakhtoura NG, Saade E, Wilson BM, Perez F, Papp-Wallace KM, Bonomo RA. 2017. A 17-year nationwide study of burkholderia cepacia complex bloodstream infections among patients in the united states veterans health administration. Clin Infect Dis 65:1327–1334.

7.	Chang T-H, Chuang Y-C, Wang J-T, Sheng W-H. 2022. Clinical characteristics and outcomes of non-cystic fibrosis patients with burkholderia cepacia complex bacteremia at a medical center in taiwan. J Microbiol Immunol Infect 55:1301–1309.

8.	Moore JE, McCaughan J, Rendall JC, Millar BC. 2024. Case report: The conundrum of what to pick? Antibiotic susceptibility variability in burkholderia cenocepacia in cystic fibrosis: implications for antibiotic susceptibility testing and treatment. Br J Biomed Sci 81:12749.

9.	Shukla A, Rodriguez S, Brennan-Krohn T. 2024. Activity of antibiotics against burkholderia cepacia complex in artificial sputum medium. J Antimicrob Chemother 79:2867–2876.

10.	Gutiérrez Santana JC, Coria Jiménez VR. 2024. Burkholderia cepacia complex in cystic fibrosis: Critical gaps in diagnosis and therapy. Ann Med 56:2307503.
